# Preliminary Efficacy Report and Prognosis Analysis of Endoscopic Endonasal Nasopharyngectomy for Recurrent Nasopharyngeal Carcinoma

**DOI:** 10.3389/fsurg.2021.713926

**Published:** 2021-08-30

**Authors:** Zhouying Peng, Yumin Wang, Yaxuan Wang, Ruohao Fan, Kelei Gao, Hua Zhang, Zhihai Xie, Weihong Jiang

**Affiliations:** Department of Otolaryngology Head and Neck Surgery, Xiangya Hospital, Central South University, Changsha, China

**Keywords:** recurrent nasopharyngeal carcinoma, endoscopic, nasopharyngectomy, survival, prognostic factors

## Abstract

**Background:** Compared with radiotherapy, endoscopic endonasal nasopharyngectomy (EEN) is increasingly used to treat recurrent nasopharyngeal carcinoma (NPC) because of its good prognosis and mild complications. This study aims to investigate the efficacy of EEN in the treatment of recurrent NPC and factors affecting prognosis.

**Methods:** This study included all patients who received EEN for recurrent nasopharyngeal carcinoma from April 2016 to April 2020. All operations were performed in Xiangya Hospital Central South University. The patient's 2-year overall survival (OS) rate, disease-free survival (DFS) rate and significant prognostic factors are reported.

**Results:** There were 38 (67.9%) males and 28 (32.1%) females, with a median age of 43 (range, 24–69 years).43 (76.8%) of the patients in our study were in advanced rT3-rT4 stage and 32 (74.4%) of the patients in the advanced stage had tumor growth closely related to the internal carotid artery (ICA). During a mean follow up period of 44 month (range 1–65 months) post-surgery. The 2-year OS rate was 48.6%, 2-year DFS rate was 42.6%. The 2-year OS rates of rT1-2 and rT3-4 recurrent NPC were 83.9 and 35.6%, respectively. The 2-year DFS rates of rT1-2 and rT3-4 recurrent NPC 76.2 and 56.3%. The advanced T stage were associated with a poor prognosis in terms of OS and DFS.

**Conclusions:** Data indicate that T staging may be an independent prognostic factor for OS and DFS. Through proper preoperative evaluation, EEN is an alternative treatment option for advanced recurrent NPC that ensures a certain level of efficacy and is relatively safe with few complications. However, additional studies with long-term follow-up and a larger sample size are required.

## Introduction

Nasopharyngeal carcinoma (NPC) has a high incidence in South Asia and China. It originates from the epithelial lining of the nasopharynx and occurs in association with EBV infection (1). NPC is sensitive to radiation therapy, and with the development of diagnostic and radiation therapy techniques, the 5-year survival rate reaches 50–64.4%, but there are still 10–20% of patients who experience regional recurrence after improvement (2, 3). Complications occurring after radiation are also a major factor that seriously affects patients' survival and quality of life (4).

The treatment of recurrent NPC is still remains challenging, and the main treatment modalities are re-irradiation, surgery or chemotherapy (4–6). Currently, re-irradiation is still the most widely used treatment modality, but the local control rate of re-radiotherapy is only 50%, and the toxic effects of re-irradiation are a concern (3). Comparing with endoscopic endonasal nasopharyngectomy (EEN), the patients with re-irradiation often suffer from severe complications such as multiple cranial nerve palsies, osteonecrosis, and internal carotid artery (ICA) dissection (2, 7, 8).

With the development of endoscopic surgical technology and the surgeon's familiarity with nasopharyngeal anatomy, EEN for recurrent NPC has become another major treatment modality (6). The development of endoscopic surgery has allowed surgeons to treat many deeply located tumors conveniently without causing any functional disabilities. There were some meta-analysis studies showed that, compare with re-irradiation, EEN showed better survival outcomes (5-year overall survival (OS) rate: 73% for EEN and 41% for reirradiation) and better quality of life for recurrent NPC (9, 10).The aim of this study was to summarize the prognosis and outcome analysis of those who underwent EEN of recurrent NPC in the past 4 years at our institution, especially for patients with local recurrence and high T-staging.

## Materials and Methods

### Patient Selection

This study included all patients who received EEN for recurrent nasopharyngeal carcinoma from April 2016 to April 2020. All patients had imaging showing the presence of recurrent NPC by nasal endoscopy, nasopharyngeal computed tomography (CT), cranial-base enhanced magnetic resonance imaging (MRI) and whole body positron emission tomography (PET) as routine and the diagnosis was confirmed by clinical endoscopic biopsies (11).

The inclusion criteria for surgery were the presence of recurrent tumor and no significant contraindication to surgery in the patient. Exclusion criteria for surgery were the presence of distant metastases or the inability of the patient to undergo surgery. All surgeries were performed by Dr. Weihong Jiang at Xiangya Hospital Central South University. Other additional clinical information was collected through medical case records and follow-up.

### Surgical Procedure

The extent of the final surgical resection, whether and how the nasopharynx needs to be reconstructed is a major concern for surgeons performing ENN, which makes preoperative evaluation particularly important. Each patient should have preoperative nasopharynx high resolution CT with thin-section and enhanced MRI of the nasopharynx to determine the exact site of recurrent tumor invasion. The whole-body PET- CT to rule out the possibility of distant metastases. If the preoperative whole-body PETCT shows high standard uptake value (SUV) in the cervical lymph nodes and a high suspicion of cancer metastasis, a lymph node dissection of the corresponding area will be performed along with ENN. The extent of surgical excision is determined by whether the soft palate, hard palate, eustachian tube, tensor veli palatini, and levator veli palatini are violated, and whether the rupture foramen area is violated. Sometimes recurrent tumor is closely related to ICA, preoperative tests related to blood vessels such as ICA intimal plaque imaging and digital subtraction angiography (DSA) should be added to determine whether ICA is invaded by tumor (12). If the ICA is only encircled by the tumor and does not break through the epicardial layer, only the epicardial stripping of the ICA should be performed to ensure the safety of the incision margin. If the ICA intima is invaded by tumors, ICA embolization or bypass surgery were performed before EEN. The line between the maxillary nerve and the pterygoid nerve can be used as a landmark to find the paraclival segment of the ICA. In these cases, the nasopharyngeal resection is usually reconstructed with temporalis flap repair because of the large extent of the nasopharyngeal resection or the accompanying ICA exposure.

### Statistical Analysis

Collect the following gender including age, gender, initial lymph node metastasis, treatment at initial presentation, time to recurrence, treatment after recurrence, lymph node metastasis at recurrence, concomitant symptoms or disease at recurrence, postoperative treatment, and survival status. The follow-up period was defined as the period from the time of first surgery in our institution to the date of death or last contact. The statistical package for the social sciences version 25.0 (IBM SPSS Statistic 25.0) was used for statistical analysis. Kaplan–Meier product-limit analysis was used to calculate the overall survival (OS) rate, disease-free survival (DFS) rate. OS rate was calculated from the date of surgery to the date of death from any cause. The log-rank (Mantel-Cox) test was used to separately test the impact of potential prognostic factors on OS and DFS rates. Then use the Cox proportional hazards model for univariate analysis. A value of P < 0.05 is considered significant.

## Results

Details of the included patients is depicted in Table 1. In total, 56 patients with recurrent NPC were identified, of which 38 (67.9%) were male and 18 (32.1%) were female. Their median age was 43 (range, 24–69 years). These patients were classified according to the American Joint Committee on Cancer-AJCC/UICC rTNM staging system (13), the tumors were staged as follows: rT1 (*n* = 9), rT2 (*n* = 4), rT3 (*n* = 26), and rT4 (*n* = 17). The number of patients with lymph node metastasis at the time of recurrence was 23 (41.1%), including 11 patients who had additional cervical lymph node dissection. A total of 11 (19.6%) patients had skull base bone or soft tissue necrosis, all of them underwent additional skull base necrotic bone or tissue removal. In 32 (57.1%) patients, the recurrent tumor was adjacent to or encircled the ICA, and 19 of these patients had preoperative or intraoperative management of the ICA. These 19 patients had preoperative enhanced MRI showing ICA encircled by tumor, and 10 patients underwent ICA embolization prior to ENN after further examination. Another seven patients tested positive for balloon embolization test (BOT), only two of whom underwent preoperative bypass surgery and both are alive to date. The remaining five patients removed the adventitia of ICA during the ENN, and three of them are still alive today. The nasopharynx was repaired and reconstructed in 27 (48.2%) patients, of these patients,16 patients underwent temporalis muscle flap repair, two patients took their own broad fascia of the thigh for repair and reconstruction, and 9 patients underwent nasal septal mucosal flap repair. Which method of nasopharyngeal reconstruction and repair is utilized depends mainly on the extent of the nasopharyngeal defect and whether the temporalis muscle flap and its blood supply vessels are invaded by the tumor. There were 19 (34.0%) patients received postoperative treatment including radiotherapy, chemotherapy, immunotherapy, or secondary surgery.

**Table 1:** Demographics and clinical characteristics of recurrent nasopharyngeal carcinoma.

**Characteristics**	**Patient number (*N*)**	**Proportion (%)**
**Gender**
Male	38	67.9
Female	18	32.1
**Age (years)**
≥50	29	51.8
<50	27	48.2
**Initial lymph node metastasis**
Yes	26	46.4
No	30	53.6
**Primary treatment**
IMRT and C	53	94.6
IMRT, C and other treatments	3	5.4
**Interval to recurrence**
≥3 years	39	69.6
<3 years	17	30.4
**Recurrent T staging**
rT1	9	16.1
rT2	4	7.1
rT3	26	46.4
rT4	17	30.4
**Other preoperative treatments**
IMRT	1	1.8
Chemotherapy	6	10.7
Immunotherapy	1	1.8
Surgery	2	3.6
C and other treatments	4	7.1
No	42	75.0
**Recurrence lymph node metastasis**
Yes	23	41.1
No	33	58.9
**Bone or soft tissue necrosis**
Yes	11	19.6
No	45	80.4
**Chronic sinusitis**
Yes	42	75.0
No	14	25.0
**Reconstruction method**
Nasoseptal flap	9	16.1
Temporalis muscle flap with tip	16	28.5
Broad fascia of thigh	2	3.6
No	29	51.8
**Cervical lymph node dissection**
Yes	11	19.6
No	45	80.4
**Postoperative adjuvant therapy**
IMRT	4	7.1
Chemotherapy	5	8.9
Immunotherapy	3	5.4
Surgery	2	3.6
C and other treatments	5	8.9
No	37	66.1
	**Total** ***N*** **=** **56**	

*IMRT, intensity-modulated radiotherapy; C, chemotherapy*.

The average follow-up time for this group of patients was 44 (44, range 1–65 months). The mean time between initial treatment and reissue of the first surgical treatment for EEN was 5.8 years. Thirty two of 56 patients (57.1%) are currently alive, including five patients with tumor, and the main causes of death include hemorrhage was six (25.0%), cachexia due to dysphagia was nine (37.5%), distant metastasis was five (20.9%), recurrence again was two (8.3%), or other causes was two (8.3%).

No patients experience acute and severe complications after surgery. The most frequent adverse symptoms presented by patients were headache (25%), nasal dryness (17.9%), difficulty in opening the mouth and swallowing (23.2%), neck swelling (10.7%), hearing abnormalities (34.0%) or occasional nasal bleeding symptoms (34.0%). However, most of the above symptoms already existed after the first radiotherapy, and it is difficult to resolve these symptoms with surgical treatment.

**Figure 1:**
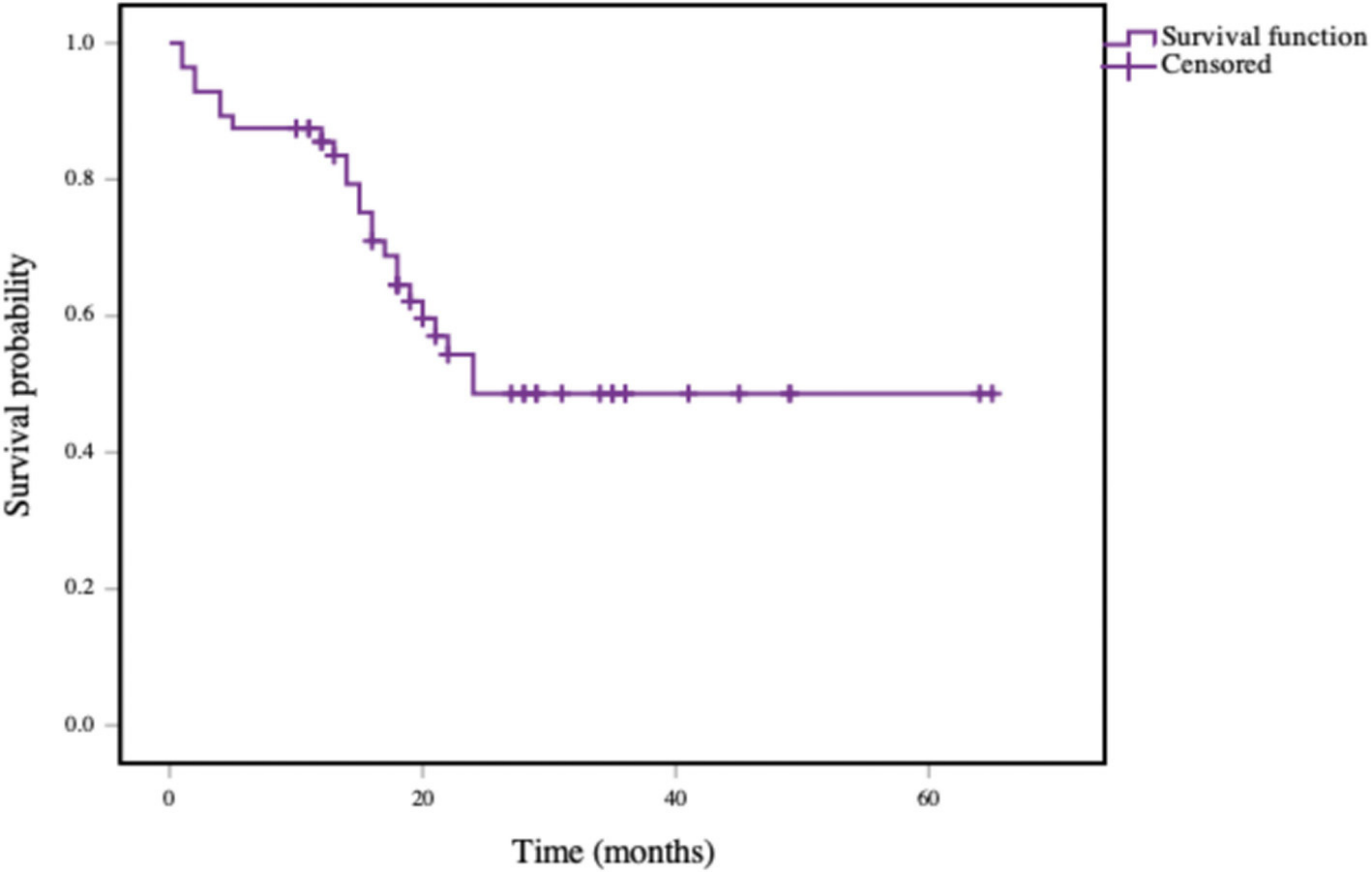
Kaplan-Meier curve of overall survival in patients with recurrent NPC.

**Figure 2:**
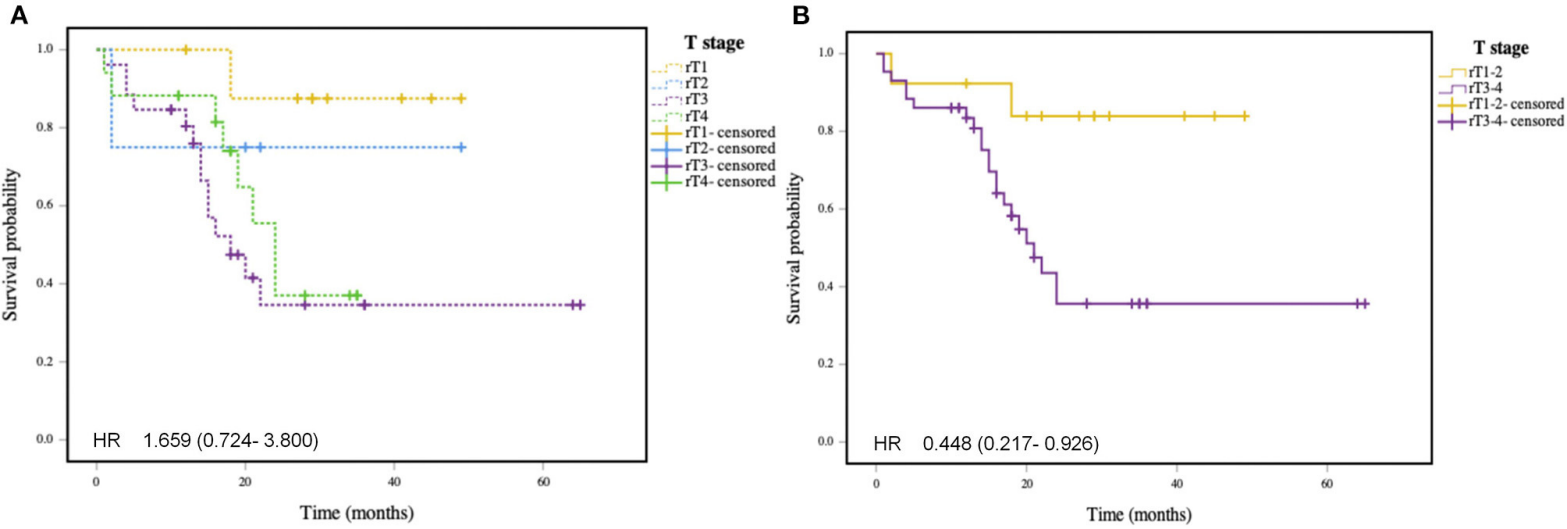
**(A)** Kaplan-Meier curve of overall survival in patients with different rT stages. **(B)** Kaplan-Meier curve of overall survival in patients with early stage recurrent NPC and advance stage recurrent NPC (*P* < 0.05).

**Figure 3:**
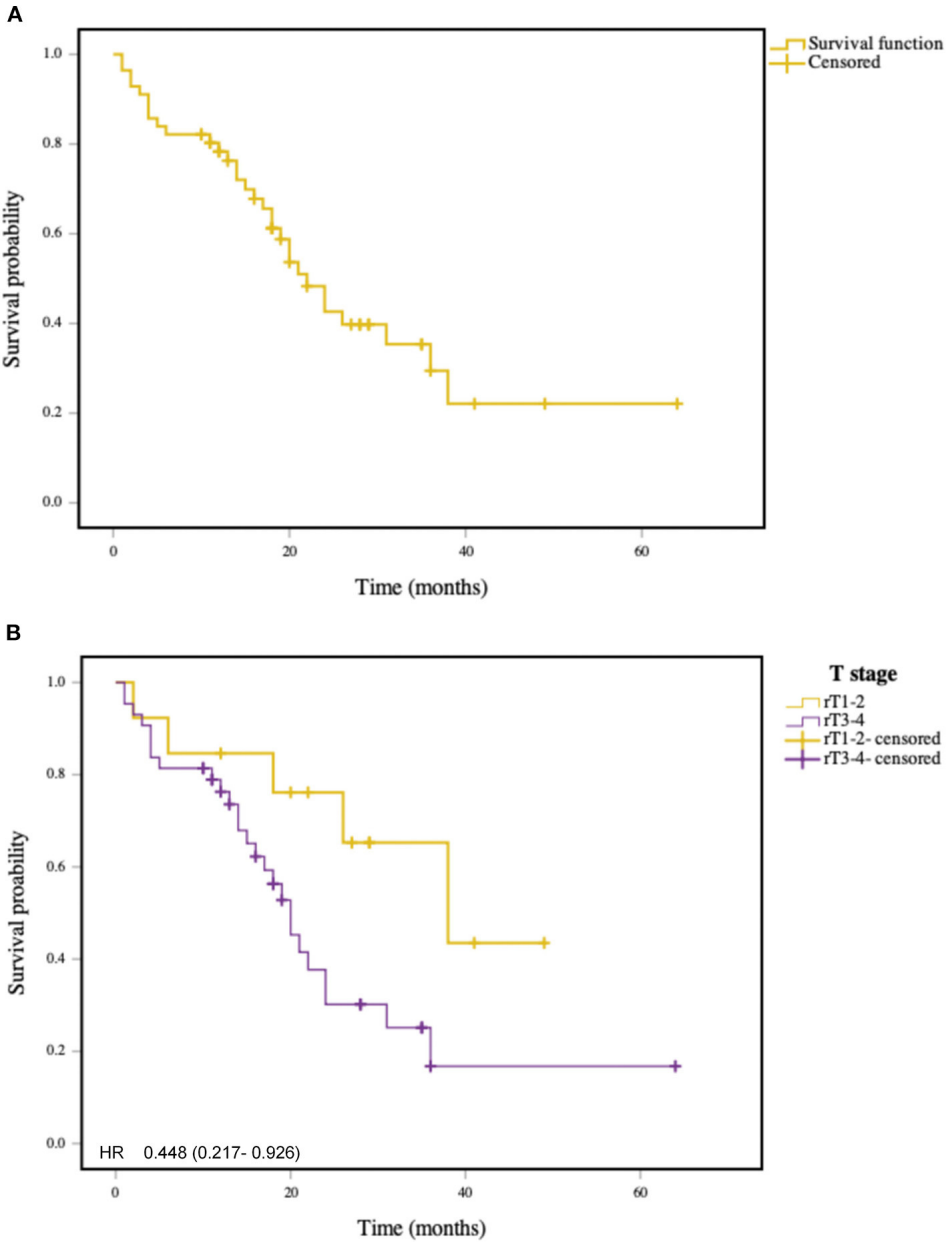
**(A)** Kaplan-Meier curve of disease free survival in patients with recurrent NPC. **(B)** Kaplan-Meier curve of disease free survival in patients with early stage recurrent NPC and advance stage recurrent NPC (*P* < 0.05).

The mean 2-year OS rate of patients was 48.6% as shown in Figure 1. The 2-year OS rates of patients with rT1, rT2, rT3, and rT4 staging tumors were 87.5, 75.0, 34.6, and 37.0%, respectively. The rT1-2 and rT3-4 staging patients had a 2-year OS rates of 83.9 and 35.6%, respectively. The mean 2-year DFS rate of patients was 42.6% (Figure 2), and the 2-year DFS rates of patients with rT1, rT2, rT3, and rT4 was 87.5, 50.0, 25.2, and 37.0%, respectively. The 2-year DFS rates of rT1-2 and rT3-4 was 76.2 and 56.3%, respectively (Figure 3).

**Figure 4:**
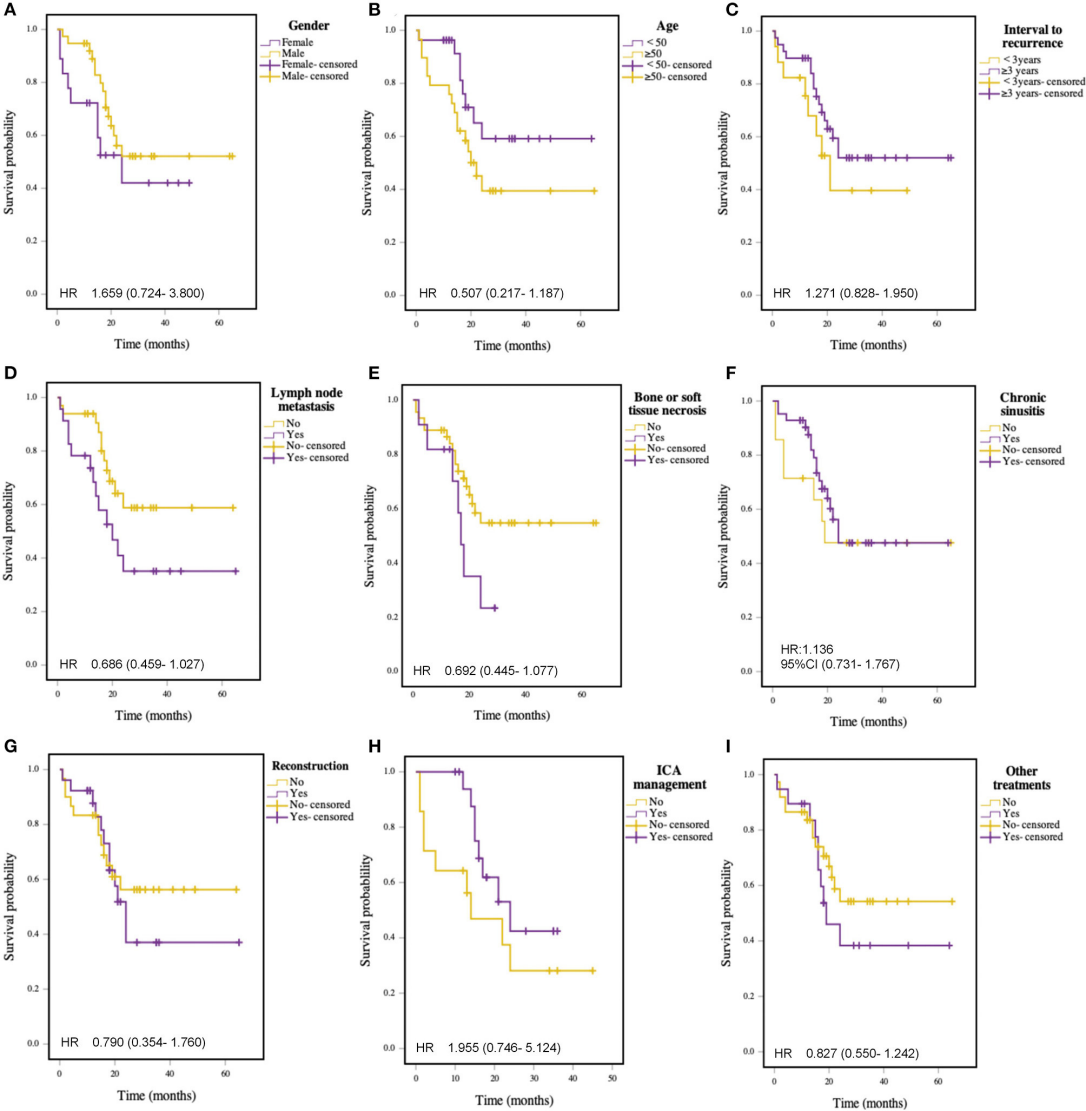
Kaplan-Meier curve for survival in patients with recurrent NPC: **(A)** Gender; **(B)** Age (≥ 50 vs. <50 years); **(C)** Interval of recurrence (≥3 vs. <3 years); **(D)** Lymph node metastasis; **(E)** Bone or soft tissue necrosis; **(F)** Chronic sinusitis; **(G)** Reconstruction; **(H)** ICA management; **(I)** Other treatments.

Univariate analysis showed a significant association between early recurrent tumors (rT1-2) and advanced recurrent tumors (rT3-4) on OS (*P* = 0.015) and DFS (*P* = 0.041). Cox regression analysis also indicated that it could be an independent prognostic factor for OS and DFS. There is no significant correlation between gender, age, time to recurrence, lymph node metastasis, bone or soft tissue necrosis, chronic sinusitis, nasopharyngeal repair and reconstruction, ICA management, and other postoperative treatments, as shown in Figure 4. The results of the analysis of individual prognostic factors are shown in Table 2.

**Table 2:** Univariate analysis of prognostic factors associated with OS and DFS in patients with RNPC.

**Variables**	**No. of patients (%)**	***P*** **-value**	
		**OS**	**DFS**
Gender		0.222	0.257
Male	38 (67.9)		
Female	18 (32.1)		
Age (years)		0.108	0.176
≥50	29 (51.8)		
<50	27 (48.2)		
Recurrence lymph node metastasis		0.058	0.309
Yes	23 (41.1)		
No	33 (58.9)		
Interval to recurrence		0.263	0.259
≥3 years	39 (70.0)		
<3 years	17 (30.0)		
Recurrent T staging		0.015	0.041
rT1-2	13 (23.2)		
rT3-4	43 (76.8)		
Bone or soft tissue necrosis		0.092	0.301
Yes	11 (19.6)		
No	45 (80.4)		
Chronic sinusitis		0.565	0.189
Yes	42 (75.0)		
No	14 (25.0)		
Reconstruction		0.559	0.866
Yes	26 (46.4)		
No	30 (53.6)		
Postoperative other treatments		0.352	0.840
Yes	19 (34.0)		
No	37 (66.0)		
ICA management		0.158	0.312
Yes	18 (56.2)		
No	14 (44.8)		

*ICA, internal carotid artery*.

## Discussion

Although NPC is a malignant tumor sensitive to radiotherapy, local recurrence or lymph node metastasis is still the main reason for failure of initial radiotherapy (3, 4). With the development of medical technology, treatment modalities for recurrent NPC are gradually enriched. In addition to traditional re-irradiation, surgical treatment, proton heavy ion therapy, targeted therapy and immunotherapy are beginning to be implemented (14). Currently, radiotherapy is still the main treatment method for recurrent NPC, but due to the high success rate and low complication rate, salvage surgery is generally recommended for recurrent NPC.

Intensity-modulated radiotherapy (IMRT) as the traditional treatment modality for recurrent NPC, has been extensively reported for its efficacy (15–19). A meta-analysis by Leong et al., which investigated 12 studies including 1,768 patients, concluded that the 5-year local failure-free survival of IMRT for recurrent NPC was 72%, 5-year distant failure-free survival was 85%, and 5-year OS was 41% (10). For patients with residual tumor or recurrence after initial treatment, the possibility of tumor resistance to radiotherapy still exists with re-irradiation with or without chemotherapy. Surgical treatment, however, offers another treatment option for these patients (4). There are many articles have been published comparing the efficacy of IMRT with EEN. According to the reported research results, surgery not only leads to similar survival and prognosis as IMRT, but also has better outcomes in terms of complications and severity compared to those who undergo IMRT (3, 7, 8).

Surgical treatment includes external approaches and transnasal endoscopic nasopharyngectomy. There are a meta-analysis study by a certain person comparing endoscopic surgery with open surgery for recurrent NPC showed that for patients with rT3 stage endoscopic surgery treatment had a 2-year OS rate look of 67% compared to 53% for open surgery and 35% for rT4 (20). Then compared with external approaches, EEN shows favorable local control and survival outcomes and is indeed a good superficial approach when considering postoperative morbidity, patient quality of life, and length of hospital stay.

Our univariate analysis showed that the T-staging of the recurrent tumor could as an independent prognostic factor for the 2-year OS and 2-year DFS rate. The 2-year OS rate of patients with early rT1-2 in this study was 83.9%, whereas the 2-year OS rate of patients with advanced rT3-4 was only 35.9% (*P* < 0.05). EEN for advanced recurrent NPC is considerably more difficult, due to some of these patients opt for surgical resection only after receiving re-irradiation, chemotherapy, or other combination treatments that have failed. At this time, the anatomical structure of the nasopharynx is poorly marked and the mucosa is scarred after radiotherapy (21, 22). The majority of patients with advanced recurrent tumor have a close relationship between their tumor growth and the ICA, or even encircle the ICA. For these cases, the surgeon is required to carefully assess whether the ICA has been violated before surgery and to discuss the surgical plan in advance. In this group of 56 patients who underwent EEN, there were 32 patients with tumor growth sites closely related to the ICA, and 21 of them had tumor encircling a segment of the artery. The imaging results were used to determine the need for preoperative embolization of the affected ICA. Before embolization of the ICA, a BOT of the artery is performed, and a negative BOT result is required before embolization is performed. For patients with ICA invasion who do not have the chance for embolization but strongly request for surgery, the ICA bypass grafting will be performed first and then EEN treatment will be performed if the patient's own conditions allow. If the tumor only encircled the ICA and did not invade its endothelial layer, only the outer membrane of the artery should be peeled off, and after the operation, the temporalis muscle flap with tissues should be repaired. The patient's own broad fascia of the thigh could be used for repair nasopharynx, if the temporalis muscle flap could not be used (involvement of the temporalis muscle or blood vessels). The 2-year OS rate was 35.4% for the 32 patients in our study whose tumors were closely related to the ICA, and the 2-year OS was 42.4% for those who underwent ICA surgery intervention and 28.1% for those who did not.

The overall 2-year OS and 2-year DFS rate in this study were lower than those currently reported in the literature (7, 8, 21–28), due to the fact that 43 (76.8%) of the patients in this group were in advanced rT3-4 stage and 32 (74.4%) of the patients in the advanced stage had tumor growth closely related to the ICA. In fact, there were not many efficacy studies focusing on advanced rT3-rT4 recurrent NPC that has been published. Wong et al., reported 2-year survival data and 5-year survival data after surgery for rT3-4 recurrent NPC patients in two publications published in 2017 and 2020, respectively (21, 22). Their results were similar to the ones we reported, with lower values compared to the results of other publications with predominantly rT1-2 recurrent NPC. However, since T-stage should be the largest factor affecting survival after surgery for recurrent NPC, our data are also not suitable for direct comparison with other papers. The prognosis of this group of patients is definitely worse than that of patients with early staging recurrent NPC. However, for these patients, EEN becomes their last choice because other treatments are ineffective. In order to bring some hope to patients with advanced recurrent NPC, surgeons should formulate strict preoperative evaluation, surgical resection and postoperative follow-up plans to maximize the survival rate of these patients and strive to improve their postoperative quality of life.

## Conclusion

In this report, a total of 56 patients with recurrent NPC who underwent EEN in the last 4 years were analyzed. Our results suggest that T staging could be an independent prognostic factor for OS and DFS. The EEN is an alternative treatment option for advanced recurrent NPC that ensures a certain level of efficacy and is relatively safe with few complications. However, additional studies with long-term follow-up and a larger sample size are required.

## Data Availability Statement

The raw data supporting the conclusions of this article will be made available by the authors, without undue reservation.

## Ethics Statement

The studies involving human participants were reviewed and approved by Xiangya hospital Research Ethics Committee of the Central South University. The patients/participants provided their written informed consent to participate in this study. Written informed consent was obtained from the individual(s) for the publication of any potentially identifiable images or data included in this article.

## Author Contributions

WJ, HZ, and ZX conceived and designed the study. ZP, YaW, RF, KG, and YuW acquired data, performed the analysis, prepared the figures and tables. ZP wrote the main manuscript. WJ supervised the study. All of the authors reviewed the manuscript. All authors read and approved the final manuscript.

## Conflict of Interest

The authors declare that the research was conducted in the absence of any commercial or financial relationships that could be construed as a potential conflict of interest.

## Publisher's Note

All claims expressed in this article are solely those of the authors and do not necessarily represent those of their affiliated organizations, or those of the publisher, the editors and the reviewers. Any product that may be evaluated in this article, or claim that may be made by its manufacturer, is not guaranteed or endorsed by the publisher.
